# Lobar Pneumonia Complicated by Pleurisy Treated with Polyvalent Serum

**Published:** 1919-01

**Authors:** 


**Lobar Pneumonia Complicated by Pleurisy Treated with Polyvalent Serum.** A. Cowan Guthrie. *The British Medical Journal*, October 19, 1918.

Though Cole contended that serum treatment would be quite useless in the state of grey hepatization in pneumonia, A. Cowan Guthrie found, on the contrary, that cures were possible, and in support of his assertion gives the history of one of his own cases.

Cole said that sterile filtered inflammatory exudates modify the course of infection. Bail thought that the hypothetical substances existing in these fluids, designated by him aggressins, were secreted by the bacilli during their growth in the animal body and acted by inhibiting or neutralizing the defensive mechanisms of the body.

Wasserman and Citror believe, on the contrary, that these aggressins merely represent bacterial substances which may go into solution either within the body or during autolysis "in vitro", and that they act by fixing the humoral immune bodies, so rendering them ineffective.

Cole's first observations were made on the fluid removed from the chests of persons suffering from empyema. Tests of the patient's blood showed that it possessed well marked protective agglutinative properties. The empyema fluid possessed no such powers.

One experiment showed the inhibiting action of empyema fluid on the agglutination of pneumococci by immune serum, and another the inhibiting action of empyema fluid on the protection of mice by immune serum. Cole considers that, as in empyema, it is practically impossible to produce a focal immunity reaction until the focus is opened up, and the fluid, with its contents of neutralizing substances, are no longer protected from the natural or artificial defensive mechanisms of the body and so may be overcome.

A child was suffering from lobar pneumonia. Patches of consolidation were found at the base of the right lung. Swabs taken from the throat teemed with pneumococci revealing a state of pneumococcemia. After an injection of 10 cc. of a polyvalent antipneumococcal serum the child regained consciousness, and temperature and pulse improved.

Two days later, the condition having become worse, a second 10 cc. of serum was injected subcutaneously and again the patient improved.

After complete removal by aspiration of the sero-purulent fluid and final drainage of the chest the child completely recovered.

